# Protein cysteine S-glycosylation: oxidative hydrolysis of protein S-glycosidic bonds in aqueous alkaline environments

**DOI:** 10.1007/s00726-022-03208-7

**Published:** 2022-12-02

**Authors:** Alicja K. Buchowiecka

**Affiliations:** Institute of Molecular and Industrial Biotechnology, Lodz University of Technology, 2/22 Stefanowskiego Street, 50-537 Lodz, Poland

**Keywords:** Proteomics, Post-translational modifications, Glycoproteins, S-linked glycosylation

## Abstract

Some glycoproteins contain carbohydrates S-linked to cysteine (Cys) residues. However, relatively few S-glycosylated proteins have been detected, due to the lack of an effective research methodology. This work outlines a general concept for the detection of S-glycosylation sites in proteins. The approach was verified by exploratory experiments on a model mixture of β-S-glucosylated polypeptides obtained by the chemical transformation of lysozyme P00698. The model underwent two processes: (1) oxidative hydrolysis of S-glycosidic bonds under alkaline conditions to expose the thiol group of Cys residues; (2) thiol S-alkylation leading to thiol S-adduct formation at the former S-glycosylation sites. Oxidative hydrolysis was conducted in aqueous urea, dimethyl sulfoxide, or trifluoroethanol, with silver nitrate as the reaction promoter, in the presence of triethylamine and/or pyridine. The concurrent formation of stable protein silver thiolates, gluconic acid, and silver nanoclusters was observed. The essential de-metalation of protein silver thiolates using dithiothreitol preceded the S-labeling of Cys residues with 4-vinyl pyridine or a fluorescent reagent. The S-labeled model was sequenced by tandem mass spectrometry to obtain data on the modifications and their distribution over the protein chains. This enabled the efficiency of both S-glycosidic bonds hydrolysis and S-glycosylation site labeling to be evaluated. Suggestions are also given for testing this novel strategy on real proteomic samples.

**Supplementary Information:**

The online version contains supplementary material available at 10.1007/s00726-022-03208-7.

## Introduction

Cellular proteins undergo many structural modifications, which determine their vital functions and activities in living organisms. Of the various functional groups in protein side chains, cysteine thiols carry the largest number of diverse post-translational modifications (PTMs). The two dominant and most studied PTMs of proteins are phosphorylation and glycosylation. Intriguingly, although they appear on numerous amino acid residues, they are only sporadically observed on protein cysteines. For a long time, S-phosphorylation (excluding the transitional process that occurs in the catalytic centers of several enzymes) and S-glycosylation were therefore considered rare and of little research interest.

The first mentions of protein cysteine S-glycosylation appeared in 1971 and referred to the peptides Gal-Gal-CEHSHDGA and Glc-Glc-Glc-Glc-CEGHSHDHGA. These peptides were isolated from male urine and human erythrocytes, respectively (Lote and Weiss 1971; Weiss et al. 1971). Only in 1998 did researchers prove that Cys26 in the heavy chain H1 of the human inter-alpha-trypsin inhibitor complex (ITI H1) is S-glycosylated by an unknown di-hexose (Olsen et al. 1998) (Olsen et al. 1998). Recently, bioactive bacterial peptides known as bacteriocins have been discussed in a series of articles. Five of these peptides are β-S-glycosylated by D-glucose (D-Glc) or N-Acetyl-D-Glucosamine (D-GlcNAc) and belong to the class of glycocins (Norris and Patchett 2016). Currently, the known *S-glycosylated glycocins* include the following: Sublancin (Oman et al. 2011; Wang and van der Donk 2011), Glycocin F (Amso et al. 2018; Bisset et al. 2018; Venugopal et al. 2011), ASM1 (PASM1) (Bisset et al. 2018; Kaunietis et al. 2019), Palladocin (Hata et al. 2010; Pranckute et al. 2015); Thurandacin A and B (Wang et al. 2014). Some studies have pointed out the interdependence between S-glycosylation and the antimicrobial activity of glycocins.

In 2016–2017, experiments using mouse and human cell proteins to investigate dynamic O-GlcNAcylation on serine and threonine unexpectedly revealed the co-occurrence of cysteine S-glycosylation by N-acetylglucosamine (S-GlcNAcylation) (Maynard et al. 2016; Xiao and Wu 2017). Moreover, protein clustering results showed that human glycoproteins modified with S-GlcNAc were mainly involved in the regulation of cell − cell interactions and gene expression (Xiao and Wu 2017). Recent advanced MS analyses have unambiguously located mono-ADP-ribosylation in protein sequences on Cys, as well as other amino acid residues (Bonfiglio et al. 2017; Buch-Larsen et al. 2020). According to the literature, protein S-glycosylation occurs in prokaryotic and eukaryotic cells. These modifications may be more widespread than evidenced by the current state of knowledge, and could even be seen as new structural elements of the sugar code system (Gabius et al. 2021).

Here, we present a concept for a tailored analytical approach enabling the detection of protein S-linked glycosylation, as a step towards the design of a general methodology. The core step is the hydrolysis of S-glycosidic bonds under mild alkaline conditions, which are not harmful to related O- and N-glycosidic linkages in glycoproteins. The approach was challenging, as in aqueous alkaline solutions both alkyl acetals and thioacetals (i.e., O- and S-glycosides) exhibit perfect hydrolytic stability. These structures have short-term chemical resistance even to strongly elevated pH and temperature conditions, which ultimately cause the β-elimination of glycans. For the first time, we show that silver ions promote the hydrolysis of S-glycosidic bonds in aqueous alkaline environments in the presence of selected organic amines. Potential applications of the discovered reaction are briefly highlighted in the end.

## Materials and methods

### Reagents

Water from a Simplicity^®^ Water Purification System, Millipore (Darmstadt, Germany), was used throughout the experiments. Spectra/Por^®^ 3 Float-A-Lyzer^®^, MWCO: 3500, Biotech Regenerated Cellulose Membranes were sourced from Spectrum Laboratories Inc. Sequencing Grade Modified Trypsin was sourced from Promega (V511A). All other reagents and solvents were purchased from Sigma-Aldrich and used as received: 3-(acrylamidopropyl) trimethyl-ammonium chloride (APTA); acetonitrile (ACN); ammonium bicarbonate; barium hydroxide; dithiothreitol (DTT); dimethyl sulfoxide (DMSO); formic acid (FA); HCl; hen egg-white lysozyme; silver nitrate (AgNO_3_); solid carbon dioxide; trifluoroacetic acid (TFA); Tris; tris-carboxymethyl phosphine (TCEP); 2,2,2-trifluoroethanol (TFE); triethylamine (NEt_3_); pyridine (Py); 4-vinylpyridine (4-VPy); urea, 1-thio-β-D-glucose sodium salt, HPTLC Kieselgel 60 F254 (Merck).

### Chemical procedures

#### Obtaining the model S-glycosylated lysozyme: L-C[S-Qat]-[Dha]-C[S-Glc]-[Side +]-COOH

**Fig. 1:**
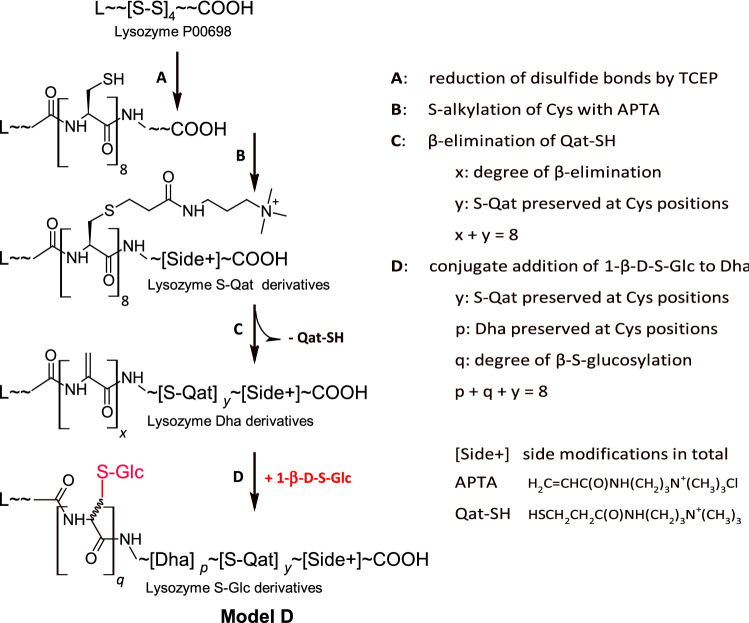
Stepwise transformation of hen egg-white lysozyme (P00698) into the model mixture of β-S-glucosylated proteins (Model D)

S-glycosylated protein was obtained by the chemical transformation of hen egg-white lysozyme P00698. The synthetic procedure comprises five basic steps and is analogous to a method described previously (Buchowiecka 2019). The three initial steps (A, B, C in. Figure 1) were the same and were performed in buffers containing 8 M urea. The procedure started with disulfide linkage reduction using TCEP (pH 5.3, 37 °C, 1 h), followed by cysteine alkylation with APTA (pH 8.1, r.t., 2 h) and β-elimination of APTA-SH (Qat-SH) using Ba(OH)_2_ (pH 12.5, 50 °C, 3 h). After each step of the transformations, the products were precipitated with an excess of isopropanol (5:1/v) and analyzed by MALDI MS. The resulting lysozyme derivatives containing on average 5–7 Dha residues per molecule, denoted as L-C[S-Qat] _2 ± 1_-[Dha] _6 ± 1_-[Side +]-COOH (Buchowiecka 2019), were subjected to Step D of the transformation.

#### Step D: Conjugate addition of 1-thio-β-D-glucose to* Dha residues*

In the next step, 180 mg of 1-thio-β-D-glucose (sodium salt) was dissolved in 20 mL of 0.2 mM water solution of Dha-containing lysozyme derivatives L-C[S-Qat] _2 ± 1_-[Dha] _6 ± 1_-[Side +]-COOH. The pH of the mixture was adjusted to 7.5 using solid TCEP, then deaerated by nitrogen sparging and incubated at 50 °C for 5.5 h. Next, the mixture was dialyzed against deionized water (3.5 kDa MWCO membrane) and freeze dried. The resulting mixture of S-glucosylated lysozyme derivatives was assigned as the model mixture or Model D.

### β-Elimination reaction on S-glucosylated lysozyme derivatives followed by fluorescent labeling of released 1-thio-β-D-glucose

Aqueous stock solutions of Model D (2 mg/mL) and barium hydroxide (4% (v/v) were prepared. Next, 75 µL of the Model D solution was combined with 150 µL of deionized water and 75 µL of the barium hydroxide solution. The resulting mixture with pH 12.4 was incubated at 50 °C for 80 min, then neutralized by adding small amounts of solid CO_2_. The rapidly precipitating barium carbonate was removed from the sample by centrifugation (5 min, 9000 g). The recovered supernatant containing 1-thio-β-D-glucose was S-alkylated with mBBr (10 µL of 100 mM mBBr in acetonitrile, r.t., 20 min in the dark). The labeling reaction was stopped by lowering the pH to 3.6 with diluted acetic acid and analyzed by TLC.

### Synthesis of fluorescently labeled 1-thio-β-D-glucose

First, 1 mg of solid 1-thio-β-D-glucose (sodium salt) was dissolved in 100 µL of deionized water (approx. pH 10) then mixed with 10 µL of an aqueous TCEP (1 mg/mL), lowering the pH to 7.9. The mixture was purged with nitrogen and incubated for 10 min at 37 °C. Next, 2 µL of 100 mM mBBr in acetonitrile was added and incubation was continued for 40 min at r.t. in the dark. The product served as a fluorescently tagged standard for TLC analysis and was applied to plates in volumes of 0.5 µL–1 µL.

### Hydrolysis of S-glycosidic bonds in Model D and consecutive labeling of cysteine thiol groups

Arbitrarily conditions for hydrolysis reactions were selected across the pH range of 7.5–9.1, measured using a Sentron 501 PocketFET pH meter (sensor type: ISFET semiconductor electrode, Sentron Inc. USA). All samples were nitrogen purged before incubation at elevated temperatures and protected from light by aluminum foil. The concentration of AgNO_3_ in each reaction mixture was 0.16 M. The final products were dialyzed against deionized water using a 3.5 kDa MWCO membrane.

### Procedure 1 in 80% aqueous DMSO

In Procedure 1, 17 mg of crystalline AgNO_3_ was dissolved in 500 µL of DMSO. The pH of the solution was adjusted to 9.1 with 0.8 µL of triethylamine. Next, 320 µL of the solution (64 µmols of AgNO_3_) was combined with 80 µL of Model D aqueous solution (3.5 mg/ml). After incubation at 50 °C for 2 h, the transparent homogenous reaction mixture gained an amber-like color, which disappeared after the addition of 9.9 mg of solid DDT (64 µmols). The instantly occurring dark precipitate was removed by centrifugation (5 min, 9000 g). The colorless supernatant with pH 2.1 was dialyzed against deionized water. The final dialysate with pH 5.1 was subjected to Cys alkylation using thiol-specific reagents.

#### Fluorescent Cys labeling with mBBr

In this step, 125 µL of the dialysate (pH 5.1), 12.5 µL of 1 M Tris–HCl buffer (pH 8.4), and 5 µL of 100 mM mBBr in acetonitrile were combined and incubated for 30 min at r.t. in the dark. The mixture was stepwise dialyzed against 10% acetonitrile (for mBBr excess removal) and deionized water. The resulting solution of labeled proteins was freeze dried, digested with trypsin, and subjected to tandem mass spectrometry by alternating CID/HCD fragmentation.

#### Cys re-blocking with APTA

In this step, 125 µL of the dialysate (pH 5.1), 12.5 µL of 1 M Tris–HCl buffer (pH 8.4), and 4 µL of 4 M aqueous solution of APTA were combined and incubated at r.t. for 30 min. The mixture was dialyzed against deionized water, freeze dried, digested with trypsin, and subjected to MS/MS analyses using both CID and HCD fragmentations.

#### Isolation of protein silver thiolates

In this step, 320 µL of 0.2 M AgNO_3_ solution in DMSO was mixed with 80 µL of Model D in water (3 mg/mL) with 1 µL of pyridine. The sample was incubated for 2 h at 50 °C in the dark. Next, 12.5 µL of the saturated water solution of NaCl was added to the mixture, causing the precipitation of AgCl, which was removed by centrifugation. The colorless transparent supernatant was treated with excess isopropanol (1:5 v/v) to precipitate putative protein silver thiolates–silver nanoclasters complexes. The separated deposit was washed with another portion of isopropanol and dissolved in an appropriate buffer before standard SDS-PAGE analysis. Under UV light, the gel displayed weak green luminescence emitted by the focused protein bands (data not shown).

##### Procedure 2 in 80% DMSO/0.4 M urea

In Procedure 2, 3.5 mg of freeze-dried Model D was dissolved in 1 mL of 2 M aqueous urea. Then, 320 mL of the solution was added dropwise to a mixture containing 1280 µL of 0.2 M AgNO_3_ in DMSO (256 µmols), 17 µL of pyridine, and 1.5 µL of triethylamine. The resulting mixture with pH 8.7 was incubated at 50 °C for 140 min. The solution gained an amber-like color, and the pH dropped to 8.1. The reaction was stopped by adding 100 µL of an aqueous solution containing 67 mg of DTT (420 µmols). The rapidly precipitating dark pellet was removed by centrifugation and the colorless supernatant with pH 3 was dialyzed against deionized water. Finally, 3 mL of the dialysate with pH 6.1 was obtained for subsequent labeling.

#### Cys labeling with 4-vinyl pyridine (4-VP)

In this step, 1300 mg of urea was dissolved in 3 mL of the protein dialysate, followed by the addition of 0.4 mL of methanol, 12 µL of 4-VP, and 1 µL of pyridine to reach pH 6.8. The reaction mixture (*v* = 4.4 mL) was incubated at r.t. for 105 min in the dark, then dialyzed against deionized water acidified to pH 4.7 with TFA. The obtained solution was freeze dried, digested with trypsin, and analyzed by MS/MS using both CID and HCD fragmentations.

##### Procedure 3 in 2 M aqueous urea

In this step, 3.5 mg of freeze-dried Model D was dissolved in 1 mL of 2 M aqueous urea. Then, 320 mL of the solution was combined with 1280 µL of 0.2 M AgNO_3_ (256 µmols) in 2 M aqueous urea and 100 µL of pyridine. After incubation in the dark at 50 °C for 140 min, the pH of the mixture dropped from 7.5 to 7.4 and the solution gained an amber-like color. The reaction was terminated by adding 100 µL of an aqueous solution containing 67 mg of DTT (420 µmols). The rapidly precipitating dark pellet was removed by centrifugation. The colorless supernatant with pH 5.8 was dialyzed against deionized water. Finally, 2 mL of the dialysate with pH 5.8 was obtained for subsequent labeling.

#### Cys labeling with 4-vinyl pyridine in 4.85 M aqueous urea

In this step, 800 mg of crystalline urea was dissolved in 2 mL of protein dialysate and combined with 0.25 mL of methanol, 8 µL of 4-VP, and 1 µL of pyridine to obtain a pH of 7. The reaction mixture (*v* = 2.75 mL) was incubated at r.t. for 105 min in the dark, then dialyzed against deionized water acidified to pH 4.7 with TFA. The obtained solution was freeze dried, digested with trypsin, and subjected to MS/MS using both CID and HCD fragmentations.

##### Procedure 4 in aqueous TFE

In Procedure 4, 35 mg of AgNO_3_ was dissolved in 300 µL of deionized water and mixed with 700 µL of trifluoroethanol, 50 µL of pyridine, and 3 µL of triethylamine to reach pH 8.6. Then, 0.5 mg of freeze-dried Model D was solubilized in 80 µL of deionized water and added dropwise to 320 µL of the alkaline silver nitrate solution (66 µmols). The sample was incubated at 40 °C for 150 min. The reaction was quenched by adding 13 mg of solid DTT (84 µmols). The mixture was centrifuged to remove the dark precipitate. The supernatant was dialyzed against deionized water with pH 4.7.

#### Cys labeling with 4-vinylpyridine

In this step, 475 mg of crystalline urea was solubilized in 0.9 mL of the protein dialysate and combined with 100 µL of methanol and 3.6 µL of 4-VP. The slight turbidity that appeared did not decrease following the addition of methanol (36 µL) or acetonitrile (50 µL). The pH of the reaction mixture was set to 6.9 using 20 µL of pyridine (*v* = 1.46 mL).Alkylation was carried out at r.t. for 60 min. The reaction was quenched by the addition of TFA, lowering the pH to 4.6. The mixture was dialyzed against deionized water of pH 4 (TFA). The resulting dialysate was freeze dried, digested with trypsin, and analyzed by MS/MS with alternating CID/HCD fragmentation.

### Analytical methods

#### TLC analysis

Appropriately diluted samples of the reaction mixtures were applied on HPTLC Kieselgel 60 F254 thin-layer plates and developed in ethyl acetate/acetic acid/water (2:1:0.5 by volume). The products were viewed under UV light.

#### Monitoring silver-promoted alkaline hydrolysis in UV/Vis absorption spectra

First, 640 µL of the alkaline silver nitrate solution (obtained according to Procedure 4) was combined with 160 µL of the Model D aqueous solution (3 mg/mL) and incubated at r.t. for 5 h in the dark. The progress of the reaction was monitored by recording the UV/Vis absorbance spectra (190–800 nm), using a Beckman spectrophotometer zeroed against a reference sample containing deionized water in place of the protein solution.

#### Mass spectrometry

#### LC-QTOF MS analysis of Model D

An aliquot of freeze-dried Model D was dissolved in 0.1% formic acid in water to prepare a 0.2 mg/mL solution. High-resolution LC/MS data were acquired using a Waters nanoAcquity UPLC system combined with a Premier quadrupole-time-of-flight (QTOF) mass spectrometer. Ten microliters of Model D solution was separated on a BEH130, C18, 75 µm × 250 mm column (Waters, nr 186,003,545). Mobile phase A consisted of 0.1% formic acid in water. Mobile phase B consisted of 0.1% formic acid in acetonitrile. The flow rate for analysis was 250 nL/min and the column temperature was 35 °C. The gradient elution program was as follows: 95% A and 5% B held at 0–1 min. The linear gradient between 1 and 160 min was 10% for A and 90% for B. The content of A was increased to 99% within 1 min and held for 5 min. The column outlet was coupled to the electrospray mass spectrometer. The MaxEnt 1 program was used to process the multiply-charged electrospray data.

#### HPLC-ESI MS/MS

A mixture of peptides was obtained from Model D by standard tryptic digestion (omitting reduction and alkylation steps). Tryptic peptides were applied to an RP-18 precolumn (Waters nanoAcquity 20 mm × 180 μm) using aqueous 0.1% trifluoroacetic acid as a mobile phase and then transferred to a HPLC RP-18 column (Waters nanoAcquity UPLC Column 250 mm × 75 μm). Chromatographic separation was performed at a flow rate of 150 nL/min using an acetonitrile gradient (0–60% in 120 min) in the presence of 0.05% formic acid. The column outlet was directly coupled to the ion source of an LTQ Orbitrap Velos (Thermo Fisher Scientific, Bergen) spectrometer working in the regime of data-dependent acquisition to register CID and HCD fragmentation spectra. The normalized collision energy was set to 30%. Dynamic exclusion was disabled. A blank run ensuring a lack of cross-contamination from previous samples preceded each analysis.

#### MS data analysis

Raw instrument data (.RAW) were processed using Byonic search engine (Protein Metrics Inc, v.0–25) against the UniProtaccession_p00698.decoys.fasta. Protein FDR was set to 1% FDR. All searches used 6 ppm precursor ion tolerance and 20 ppm fragment ion tolerance. All searches considered tryptic peptides with a maximum of two missed cleavages. The algorithm allows the selection of up to four common modifications and only one rare modification per peptide. The following variable modifications were assigned as common (c) and rare (r):

Qat/ + 170.1419 @C (c4).

Dha/ − 33.9877 @C (c4).

Hex/ + 162.052824 @C (c 4).

Pyridylethyl/ + 105.057849 @C (c4).

Bromobimane / + 190.074228 @ C (c4).

Deamidation/ + 0.9840 @N (c3).

Deamidation/ + 0.9840 @Q (c1).

Carbamylation/ + 43.0058 @R (c3).

Carbamylation/ + 43.0058 @K (c3).

Carbamylation/ + 43.0058 @NTerm (c1).

Oxidation/ + 15.9949 @M (c1).

Dioxidation/ + 31.9898 @M (c1).

Dioxidation/ + 31.9898 @W (c1).

Trioxidation/ + 47.9847 @C (c1).

Gln > pyro-Glu/ − 17.0265 @NTerm Q (r1).

Glu > pyro-Glu/ − 18.0105 @NTerm E (r1).

## Results

### Synthesis and characterization of model D

The initial research phase required inventing a feasible procedure for synthesizing S-glycosylated model proteins. The chemical transformation of lysozyme P00698 into multi-dehydroalanine-containing derivatives, earlier described in (Buchowiecka 2019), followed by the conjugate addition of 1-thio-β-D-glucose to dehydroalanine (Dha) residues, permitted the incorporation of β-S-glucosylated cysteines into the lysozyme sequence (Fig. 1). The resulting Model D was then characterized by LC–ESI–MS and MS/MS-peptide mapping to obtain data on the types of introduced modifications and their distributions within model (poly)peptides.

Native lysozyme containing four disulfide bridges was submitted to a four-step process of transformation under denaturing conditions. Step A was typical reduction of disulfide bridges. In Step B, permanent protection of cysteine sulfhydryls using APTA reagent produced a mixture of proteins S-alkylated with quaternary amine (Qat). In Step C, β-elimination of Qat-SH upon barium hydroxide led to products of the general formula L-C[S-Qat] _2±1_-[Dha] _6±1_-[Side +]-COOH carrying on average *x* = (6 ± 1) Dha residues per lysozyme molecule. Unintended side modifications [Side +] also occurred, such as deamidation of Asn and Gln or carbamylation of Lys and N-terminal amine groups (Buchowiecka 2019). The final conjugate addition of 1-β-S-Glc to the Dha produced a mixture of polypeptides of the general formula L-C[S-Qat] _2±1_-[Dha] _4±1_-C[S-Glc] _2±1_-[Side +]-COOH—i.e., bearing on cysteine positions C[S-Qat], [Dha], and C_*D/L*_[S-Glc] (Step D). The approximate degree of lysozyme S-glucosylation was determined based on the deconvoluted MS spectrum of Model D, indicating that the main products bear (2 ± 1) C[S-Glc] units per 8 cysteine positions in the lysozyme sequence. The profile of the MS spectrum also revealed an abundance of protein Lys carbamylation and Asn/Gln deamidation. A more detailed description of these results is presented in the supporting materials [ESM_Fig. S2–S4]. Minor products of polypeptide degradation due to the strongly alkaline milieu of the β-elimination reaction were detected by SDS PAGE (data not shown).

Model D was subjected to standard tryptic digestion followed by MS/MS-peptide mapping with concomitant CID and HCD spectra acquisition (Dayon et al. 2010). This enabled the construction of a spectral library of 3319 items. The collection covers 320 unique sequences, including 244 spanning Cys positions. Sixty S-hexosylated peptides C[S-Hex] = C[β-S-Glc] and 14 peptides containing free cysteines C[S–H] or cysteine oxidized to cysteic acid [CysA] = C[SO_3_H] were identified (Table 1, Model D). The number of identified unique peptides does not mirror the isomeric and isobaric complexities of the model proteins. The exemplary sequence NLCNIPCSALLSSDITASVNCAKKIVSDGNGMNAWVAWR, covering three cysteine positions and five asparagine positions, might theoretically exist in hundreds of variants, differing in terms of the distributions of five potential deamidation sites on asparagines and the following modifications on cysteines: C[S-Qat], [Dha], C_*L*_[S-Glc], C_*D*_[S-Glc], C[S–H], C[SO_3_H]. S-glucosylation occurred in all eight racemized D/L-cysteine positions (designated as C_*D*_ and C_*L*_) in the lysozyme derivatives. The MS/MS spectra of S-glucosylated peptides show the presence of multiple fragment ions carrying C[S-Glc] units, indicating the moderate tendency of this modification towards neutral glucosyl loss during CID/HCD-type fragmentation. That allowed the unambiguous assignation of S-glucosylated sites. Exemplary MS/MS spectra of the peptides are accessible in [ESM_Fig. S17-20]. Interestingly, the manual analysis of the spectral library provides evidence for a multisite gas-phase glucosyl transfer phenomenon. Therefore, a detailed description of these results will be published separately.

**Table 1:** Statistical outcomes from spectral counting of peptides (from MS/MS data) and counting the number of targeted modifications located at Cys sites in peptides

Tryptic peps. collection	MS/MS-peptide spectral counting				Numbers of modifications at C sites of unique peptides				
	total	unique	modified	with C sites	C[S-Glc]	C[S-Tag]	C[S–H]	C[SO_3_H]	C[S-Mod]
Model D	CID/HCD: 3319	313	280	244	62	114	13	3	Fig. S8
Procedure 1									Fig. S6
S-BM tagging	CID: 6272	591	544	185	109	57	55	13	Fig. S9
S-Qat reblock	CID: 6323	526	477	411	70	247	16	13	Fig. S10
S-Qat reblock	HCD: 7256	475	420	369	76	227	22	4	Fig. S11
Procedure 2									
S-PE tagging	CID: 3337	335	304	193	37	47	58	12	Fig. S12
S-PE tagging	HCD: 2298	295	262	162	29	26	23	6	Fig. S13
Procedure 3									Tab. S2
S-PE tagging	CID: 1728	449	410	259	100	73	115	124	Fig. S14
S-PE tagging	HCD: 1098	221	188	117	26	7	7	2	Fig. S14
Procedure 4									Fig. S7
S-PE tagging	CID/HCD: 4359	490	459	389	79	49	62	8	Fig. S16

Fairly deep insight into the structural complexity of Model D was achieved. This was possible due to the initial prerequisites for the transformation—i.e., the use of a single protein with a known sequence and a defined monosaccharide glycan. Given the structures of known S-linked glycans in natural proteins, S-glucosylated Model D appeared a suitable object for further investigations.

### Behavior of model D under aqueous alkaline conditions favoring β-elimination

Model D was subjected to conditions favoring β-elimination (pH 12.4; 50 °C; 80 min.), with Ba(OH)_2_ as a strong base. The eliminated glycans were labeled using the fluorescent reagent mBBr (monobromobimane). TLC analysis of the reaction products confirmed the presence of a single fluorescent bimane-labeled compound, identical to the earlier synthesized standard—i.e., BM-labeled 1-thio-β-D-glucose [EMS_Fig. S1]. This result proved the presence of β-S-glucosylation at cysteine positions in the modified lysozyme. This conclusion agrees with other findings that nucleophilic substitution and conjugate addition reactions proceed with retention of the anomeric configuration of 1-thio-β-D-glucose (Galonić et al. 2003; Wang and Zhu 2014). Other researchers have performed similar β-elimination of 1-thio-β-D-glucose in the presence of sodium borohydride (NaBH_4_) to determine the location of the S-glycosylation site in Sublancin (Oman et al. 2011).

### Behavior of model D under alkaline aqueous conditions in the presence of silver ions

Model D served to elaborate reaction conditions for specific hydrolysis of S-glycosidic bonds. In general, protein glycans are prone to hydrolysis catalyzed by protic and Lewis acids, but are stable in alkaline environments. Therefore, the intended specific S-deglycosylation promoted by thiophilic Lewis acids should proceed under alkaline conditions. Several publications (Haginoya et al. 2006; Hojo and Nakahara 2007; Katayama et al. 2010; Luhowy et al. 1973; L. Zhang and Tam 1999) decisively informed four experiments in the frames of Procedures 1–4. The proposed approach comprises two processes: (1) hydrolysis of S-glycosidic bonds under alkaline conditions to expose the thiol group of Cys residues; (2) thiol-S-alkylation leading to S-tagging of the S-glycosylation sites. These exploratory reactions were run in aqueous urea, DMSO, or TFE in the presence of selected Lewis bases: triethylamine (NEt_3_) and pyridine (Py). Silver nitrate AgNO_3_ served as a promoter for S-glucosidic bonds hydrolysis, leading to the formation of protein cysteine silver thiolates, which were next demetalated by DTT reagent. To confirm effective deglycosylation, freed Cys residues were S-alkylated by selected thiol-specific reagents, which introduced the bimanyl (S-BM), pyridylethyl (S-PE), or quaternary amine (S-Qat) labels, respectively.

Some physicochemical attributes of these labels meet the requirements of different research methodologies. Bimanyl (BM) is a hydrophobic fluorescent label introduced with monobromobimane (mBBr) at pH 8.5. The diagnostic immmonium ion 266.6 Da may appear in the MS/MS spectra of BM-tagged peptides (Petrotchenko et al. 2006). The PE-label can be introduced to peptides with 2- or 4-vinylpyridine (2-VP or 4-VP), even in an acidic milieu of pH 4.5 or higher (Ishimizu et al. 1996). In peptide fragmentation spectra, the PE-label often produces the PE^+^ reporter ion (106 Da) (Arrigoni et al. 2006). The S-Qat label provides peptides with a permanent positive charge, facilitating MS-sequencing of molecular ions of higher charge states (e.g., z =  + 3 to + 5; ESM 1–6.xlsx files). Signals of APTA loss from a molecular ion (e.g., [MH_2_-APTA] ^++^) might be registered in CID/HCD spectra, while strong 117 Da and 205 Da reporter ions can appear in ETD spectra (Vasicek and Brodbelt 2009). Beneficial features of peptides carrying C[S-Qat] or C[S-PE] tags are that they exhibit affinity to cation exchangers and can be isolated from complex samples (Winnik 2005).

The products of Procedures 1–4 performed on Model D were subjected to standard trypsinolysis, followed by MS/MS-peptide mapping. Next, the modifications located at each of eight cysteine positions of the S-labeled polypeptides were counted. This enabled a statistical representation of the particular modifications associated with the process of S-glycosylation site tagging (i.e., C[S-Glc], C[S–H], C[SO_3_H], C[S-Tag]). Table 1 presents these outcomes, along with references to appropriate supporting materials.

Identification of S-labeled sites (C[S-Tag]) and restored cysteine residues (C[S–H]) proves that splitting of S-glycosidic bonds took place. However, the presence of preserved S-glucosylated sites (C[S-Glc]) indicates that neither hydrolysis nor S-tagging (S-labeling) reached completion.

### Efficacy of S-glycosylation site detection

The efficiency of Procedures 1–4 was estimated based on analysis of qualitative MS data (Table 2). The approach relied on the counting of respective cysteine modifications within unique peptides (*i.e., modification sampling*) and the following reasoning. The sum of the detected structural units—i.e., preserved C[S-Glc] + C[S–H] + C[SO_3_H] + C[S-Tag]—is considered equivalent to the initial number of C[S-Glc] sites in Model D. The total count of identified C[S–H] + C[SO_3_H] + C[S-Tag] sites represents the fraction of S-glycosidic bonds that were hydrolyzed in the first step of each procedure. Similarly, the yield of the S-labeling step can be calculated by dividing the number of C[S-Tag] sites by the total number of hydrolyzed C[S-Glc] sites. The level of C[SO_3_H] unit formation was also evaluated. The following is an example of the calculation of fluorescent labeling of S-glucosylation sites according to Procedure 1 (see, Table 1; S-BM tagging data).

**Table 2:** Experimental conditions for hydrolysis of S-glycosidic bonds promoted by AgNO_3_, with approximate yields [%] of C[S-Glc] site hydrolysis and Cys S-tagging (based on CID data from Table 1)

Proc. #	Solvent	Base	pH	T [°C]	time [min]	# of C[S-Glc] sites in unique peptides (*CID data)*			
						Initial	Preserved	Hydrolyzed	S-tagged
**1**	80% aq. DMSO	NEt_3_	9.1	50	120	234	109; 47%	125; 53%	57; 46%
**2**	80% aq. DMSO/0.4 M urea	NEt_3_ and Py	8.7	50	140	154	37; 24%	117; 76%	47; 40%
**3**	2 M aq. urea	Py	7.5	50	140	412	100; 31%	312; 76%	73; 23%
**4**	50% aq. TFE	NEt_3_ and Py	8.6	40	150	198	79; 40%	119; 60%	62; 52%

Initial number of C[S-Glc] sites: 234 = 109 + 57 + 55 + 13.

Step 1) number of hydrolyzed C[S-Glc] sites: 125 = 57 + 55 + 13.

Step 2) number of S-labeled C[S-Glc] sites: 57.

Yield for step 1): (125/234) × 100% = 53%

Yield for step 2): (57/125) × 100% = 46%

Overall yield for steps 1) and 2): (57/234) × 100% = 24%

Yield of C[S-Glc] conversion into C[SO_3_H] (13/234) × 100% = 5.5%

Four combinations of alkaline aqueous solutions containing urea and DMSO or TFE co-solvents were prepared for the reaction of S-glycosidic bond hydrolysis. Since protein-DMSO and protein-TFE interactions are complicated (Chan et al. 2017; Kundu and Kishore 2004; Tjernberg et al. 2005), as is the chaotropic behavior of urea, the potential impact of these factors on hydrolysis was not considered.

In Procedure 1, thioglycoside bond hydrolysis was performed in 80% aqueous DMSO with critical NEt_3_ and 0.16 M AgNO_3_ additives (2 h, 50 °C, pH 9). The progress of the reaction was manifested by a gradual change from a colorless solution to a transparent amber-like colored liquid. The UV/Vis absorption spectrum of the final reaction mixture exhibited a distinct, broad absorption band with a maximum of 431 nm [ESM Fig.S6]. The subsequent addition of DTT caused de-metalation of the protein solution, which was further S-BM-tagged and subjected to MS/MS-peptide mapping. For this analytical variant, as many as 6272 CID spectra were acquired (Table 1). Out of 591 unique peptides that were identified, 185 carried one or more cysteine positions per peptide sequence. The moderate efficiency of alkaline hydrolysis (53%) and S-tagging (46%) correlates with the number of S-glucosylated sites that survived Procedure 1 and the number of C[S–H] sites that escaped labeling or were oxidized to cysteic acid. Nonetheless, S-BM-tagging identified eight S-glucosylation sites distributed over locations C6, C30, C64, C76, C80, C94, C115, and C127 in Model D. The second variant of Procedure 1 was performed to test the re-blocking efficacy of hydrolytically restored Cys thiols using APTA reagent. However, due to unoptimized re-blocking conditions Cys residues were still detectable in the analyte (Table 1).

The detection of S-glycosylation sites performed on Model D according to Procedures 2–4 generally confirmed the outcomes of Procedure 1. However, interesting results were observed for Procedure 3. In this case, S-glycosidic bonds hydrolysis was performed in 2 M urea at 50 °C and pH 7.5, with pyridine as the organic base. A survey of Cys modifications in unique peptides identified by CID gave the following results: 100 preserved S-glucosylated Cys positions, 73 S-tagged sites, 115 sites with restored free cysteine, 124 sites occupied by cysteic acid (Table 1). Thus, of the 412 initial C[S-Glc] sites, 312 were hydrolyzed, but as many as 40% (124/312) underwent irreversible oxidation, decreasing the overall tagging result. Similar estimates for other procedural variants gave a several-fold lower level of oxidized thiols (Table 3).

**Table 3:** Approximate yields of C[S-Glc] sites conversion into cysteic acid residues C[SO_3_H] in Procedures 1–4 and overall outcomes of S-glucosylation site labeling in Model D (based on CID data)

Procedure	Solvent	Initial # of C[S-Glc]	Detected # of C[SO_3_H]	Cys side oxidation level [%]	Detected # of C[S-Tag]	Overall tagging yield [%]
1	80% aq. DMSO	234	13	5.5	57	24.4
2	80% aq. DMSO/0.4 M urea	154	12	7.8	47	31.5
3	2 M aq. urea	412	124	30.1	73	17.7
4	50% aq. TFE	198	8	4.0	62	31.3
Model D	NA (not applicable)	192	3	1.5	NA	NA

## Discussion

The task of executing hydrolysis of thioacetals in an aqueous alkaline milieu required finding the adequate Lewis acid, which would remain stable and active at procedural conditions. Silver ions are prone to interaction with amino acids, proteins, numerous complexing structures, and diverse cysteine protecting groups (Eckhardt et al. 2013). In theory, silver ions satisfy two of the required features: they exhibit high thiophilic activity and oxidize reducing sugars, such as released glycans, to stable aldonic acids. However, silver ions are also prone to rapid hydrolysis in aqueous alkaline hydroxide solutions. Careful literature research for evidence on the thiophilic activity of silver ions in alkaline buffers was conducted prior to lab experiments. An early article on silver-promoted hydrolysis of thiazolidines provided a good foundation (Luhowy et al. 1973). The study demonstrated that silver cations, even when tightly complexed by protective thiosulphate ions, do retain their promoting properties in 0.05 M NaOH in the presence of NaClO_4_ ions. Other investigations describing the synthesis of glycoproteins also provided noteworthy results. Katayama et al. (2010) performed sequential coupling of N- and O-glycosylated peptides, which carried Cys residues protected by acetamidomethyl groups C[S-Acm]. The subsequent metal ion-assisted deprotection step routinely proceeds in acidic environments, which are harmful to the saccharide chains (Spears et al. 2021). To avoid this unwanted effect, the Katayama et al. were able to deprotect C[S-Acm] in aqueous DMSO in the presence of N, N-diisopropyl-N-ethylamine (DIEA), with AgNO_3_ as a reaction promoter. Another publication described silver ion-assisted lactamization and lactonization of peptide C-terminal thioesters. Both reactions are favored at pH 5–5.7 but also proceed in the range of pH 7–8, particularly in aqueous DMSO. At pH higher than 8, the precipitation of silver oxides was observed (Zhang and Tam 1999). Similarly, in studies on synthesizing complex glycopeptide sequences, silver-promoted peptide thioester coupling was accomplished in aqueous DMSO/DIEA (Haginoya et al. 2006; Hojo and Nakahara 2007). It was therefore concluded that appropriate complexing ligands can protect silver ions from hydrolysis in an alkaline milieu without blocking their thiophilic activity.

**Scheme 1:**
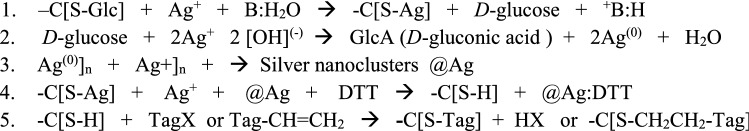
Postulated series of chemical conversions co-occurring with the main reaction of S-glycosidic bond hydrolysis promoted by silver ions. -C[S-Glc]: S-glucosylated Cys positions in the sequence of S-glucosylated lysozyme. -C[S–Ag]: cysteine silver thiolate positions in the sequence of S-glucosylated lysozyme. -C[S–H]: free cysteine positions / demetallated protein silver thiolate positions. @Ag:DTT: dithiothreitol-silver ion/silver nanocluster complexes. TagX / Tag-CH = CH_2_: Tagging reagents of alkyl halide type / vinyl type. -C[S-Tag] and -C[S-CH_2_CH_2_-Tag]: S-tagged Cys positions. B: organic amines; triethylamine and pyridine

Based on the literature, exploratory experiments on Model D were performed to test the possibility of hydrolysis of S-glycosidic bonds in aqueous alkaline environments. All procedural variants of the executed reactions gave comparable and satisfying results, which allowed us to postulate a chain of chemical conversions parallel to and triggered by hydrolysis of thioglycosidic bonds (Scheme 1).

In aqueous DMSO or TFE, Ag^+^ ions form complexes with accessible Lewis nitrogen bases and organic solvents. They also interact with numerous coordination sites in protein molecules. Once the thiophilic Ag^+^ ion approaches the S-glycosidic bond, the bridging sulfur gains a positive charge, inducing C-S linkage polarization. Next, the S-glycosidic linkage undergoes splitting by the water-amine complex B:H_2_O via S_*N*_1-like or S_*N*_2-like mechanisms (Bohé and Crich 2011). This causes the release of the protein silver thiolate C[S–Ag] and *D*-glucose (Scheme 1.1). Surrounding Ag^+^ ions enable in situ oxidation of *D*-glucose to the stable *D*-gluconic acid, with a parallel reduction of Ag^+^ to Ag^0^ (Scheme 1.2) (Chevallet et al. 2008; Sadeghi and Hosseinpour-Zaryabi 2020). The whole process can be termed the *oxidative hydrolysis* of S-linked glycans.

Co-existing silver atoms Ag^0^ and silver cations Ag^+^ give rise to the formation of small neutral and cationic silver nanoclusters @Ag (Scheme 1.3). Their presence is manifested by the appearance of a characteristic surface plasmon resonance band centered at 431 nm in the absorption UV/Vis spectrum [ESM_Fig. S6-S7] (Xie et al. 2020). Silver nanoclusters @Ag likely experience sequestration by protein silver thiolates (Y. Zhang et al. 2021). In the final stage of the procedures, protein silver thiolates -C[S–Ag] and silver nanocluster-containing proteins -C[S–Ag@Ag] undergo demetallation by DTT (Scheme 1.4), followed by S-tagging of Cys residues with selected reagents (Scheme 1.5). The addition of DTT rapidly acidifies the reaction milieu. The pH decrease favors dissolution of silver nanoclusters @Ag and the formation of reactive oxygen species (ROS) (He et al. 2011, 2012; Rong et al. 2018). The complicated redox processes, involving silver ions and silver nanoclusters, might explain the enhanced oxidation of Cys, Met, and Trp residues (Bellmaine et al. 2020; Llopis et al. 2021) observed in tryptic peptides originating from Procedures 1–4, compared to the level of Model D oxidation (ESM_Table S2). This phenomenon is particularly evident in the data for Procedure 3 executed in 2 M aqueous urea, which likely favors ROS-related oxidation as reported elsewhere (Yang et al. 2010).

The unintentional irreversible oxidation of cysteine thiols labels S-glycosylation sites by cysteic acid (CysA). However, some proteins might carry inherent CysA residues originating from intracellular processes involving ROS activities (Paulech et al. 2015). Silver-promoted hydrolysis of modifications linked to proteins by thioester bonds (e.g., Cys-palmitoylation) might also lead to false-positive S-tagging (L. Zhang and Tam 1999).

As proposed in Scheme 1, silver-promoted hydrolysis triggers the oxidation of released D-glucose to the D-gluconic acid, which is inactive in Maillard reactions and does not form D-gluconic acid 1, 4-lactone (GDL) in an alkaline milieu. This prevents proteins from glycation (Kutzli et al. 2021), as well as from N-terminal or internal gluconoylation by GDL (Martos-Maldonado et al. 2018. Under alkaline conditions gluconic acid molecules (GlcA) strongly coordinate silver ions and silver nanoclusters (Osorio-Román et al. 2011; Sadeghi and Hosseinpour-Zaryabi 2020; Sawyer 1964). The preplanned profiling of aldonic acids, resulting from oxidative hydrolysis of protein S-linked glycans, should be preceded by the removal of silver ions, silver clusters, and protein components from the reaction mixture. Some examples of aldonic acid profiling have been described previously (Larcher et al. 2009; Larsson and Samuelson 1977).

The outcomes of model Procedures 1–4 showed that the key transformations—i.e., hydrolysis of S-glycosidic bonds and S-tagging of restored Cys thiols—are fairly efficient but not quantitative. Therefore, the processed sample would likely contain proteins carrying both S-tagged and intact S-glycosylation sites, along with proteins bearing Cys residues that escaped S-tagging or were oxidized to cysteic acid. Well-elaborated S-protection operations precede most regular mass spectrometric or electrophoretic analyses to prevent unwanted intra- and intermolecular reconstruction of disulfide bonds. Thus, in some cases the reappearance of Cys residues may require repeating the S-alkylation step (i.e., a re-blocking step, as demonstrated in Procedure 1 using APTA, Table 1). The efficiency of protein Cys S-alkylation reaches at best 99% (Mouchahoir and Schiel 2018; Müller and Winter 2017; Wu et al. 2017)*,* however at the cost of random over-alkylation of protein nitrogen sites, when using haloacetamides. Nowadays, even traces of under-alkylated or reappeared Cys thiols are detectable at the MS/MS level (Geiszler et al. 2021). Recent articles on LC–MS/MS-peptide mapping of the NISTmAb protein (Mouchahoir and Schiel 2018) and other commercially available monoclonal antibody standards (Robotham and Kelly 2019) show that the complete protection of protein Cys thiols is achievable.

Applied in these studies, Model D contains D-glucose S-linked to polypeptide backbones. At present, among known S-glycosylated proteins predominate those bearing mono-ADP-S-ribosylation and Cys[S-GlcNAc] units. Referring to the latter type of modification, the outcomes of silver-promoted oxidative hydrolysis would require separate investigations in terms of a glycan part. Theoretically, the reaction might liberate S-glycans in the form of reactive glycosyl oxazolines (Fairbanks 2018) amenable to chemical catching, which would allow for further S-glycan profiling.

### Concluding remarks

The article has described the specific oxidative hydrolysis of S-glycosidic bonds promoted by Ag + ions in mild alkaline aqueous environments, which are not harmful to protein O- and N-linked glycosylation. This novel reaction leads to the formation of protein silver thiolates with the release of glycan aldehydes, in situ oxidizing to stable aldonic acids suitable for isolation and further profiling. Experimentally tested in several variants, the two-stage transformation eventually enables S-tagging of the silver thiolate sites by analytically convenient reagents. The moderate yields of both hydrolysis and S-glycosylation site tagging are not disadvantages. The moderate yields open the way for the parallel identification of proteins carrying unaffected S-glycans and their cognate S-tagged forms. Enriched fractions of glycoproteins with small-size glycans (Riley et al. 2021a) or single glycoproteins, such as human ITI H1 with still unknown di-hexose glycan, could be suitable objects for implementing the invented reaction in real-world studies.

**Fig. 2:**
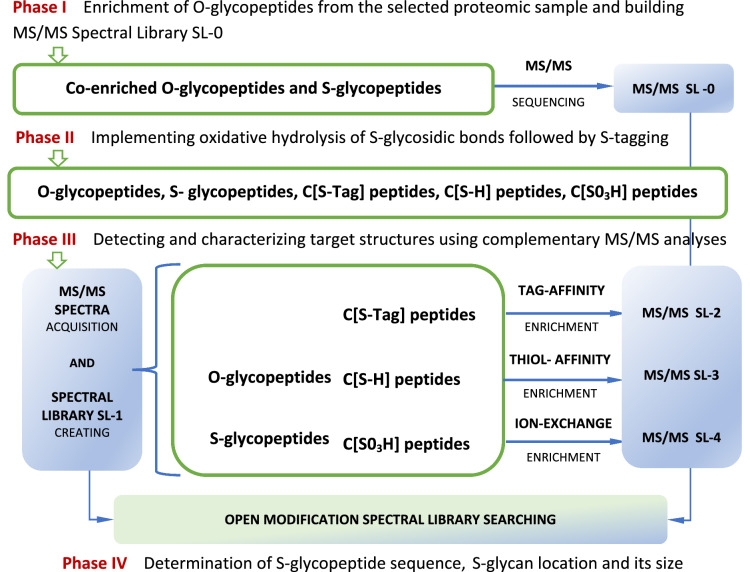
The general concept for incorporating S-glycosidic bond oxidative hydrolysis into glycoproteomic workflows

Protein S-glycans can be viewed as close structural analogs of respective O-glycans yet linked to polypeptide backbones through a more stable, isosteric S-glycosidic bond (de Leon et al. 2017). It is meaningful that during investigations on protein O-GlcNAcylation, the S-GlcNAcylated structures have also been discovered (Maynard et al. 2016; Xiao and Wu 2017). Accordingly, it deems reasonable to include research on S-glycosylation in well-developed workflows of mass spectrometry-based glycoproteomics (Bagdonaite et al. 2022; Darula and Medzihradszky 2018; Riley et al. 2021b), e.g., in the manner sketched in Fig. 2.

A mixture of enriched O-glycopeptides, attained by a suitable proteomic strategy, could be the sample for further testing. In light of the structural complexity of O-linked glycosylation, it seems unlikely to discern a presumably low level of S-glycopeptides in the initial analyte. Possibly, except for the more abundant S-GlcNAc-modified sequences, as explained earlier. Processing the analyte, first by oxidative hydrolysis liberating cysteine thiols from the peptides' S-linked glycosylation, followed by S-tagging and the LC–MS/MS analysis would lead to spectral library SL-1, as well as libraries SL-2, SL-3, SL-4 originating from suitably enriched fractions of the analyte. Open modification spectral library searches (Polasky et al. 2020) applied to the elaborated MS/MS data should enable determining the S-glycopeptides’ sequences, the S-glycans’ locations and their size.

Alternatively, proteins’ fluorescent S-glycosylation site tagging followed by simple SDS-PAGE analysis could enable fast, targeted sample screening. Once detected, the proteins could be submitted to more advanced procedures, including 2D gel-based proteomics for PTM investigations (Curreem et al., 2012; Marcus et al., 2020). It may also be beneficial to suppress ROS activity, which accompanies oxidative hydrolysis.

To put the finishing touches, it is worth reminding that O,O-acetals and O,S-acetals, with their alkaline stability, serve as fundamental structural types of diverse protecting groups utilized in synthetic organic chemistry. Therefore, the oxidative hydrolysis of sugar thioacetals in alkaline conditions might catch the attention of a broader range of scientists.

## Supplementary Information

Below is the link to the electronic supplementary material.

Supplementary file1 (DOCX 2486 KB)

Supplementary file2 (XLSX 143 KB)

Supplementary file3 (XLSX 36 KB)

Supplementary file4 (XLSX 326 KB)

Supplementary file5 (XLSX 41 KB)

Supplementary file6 (XLSX 51 KB)

Supplementary file7 (XLSX 91 KB)

## Data Availability

The author declares that the data supporting the findings of this study are available within the article and in its supplementary information files.
