# Supplementary material for: Protein cysteine S-glycosylation: oxidative hydrolysis of protein S-glycosidic bonds in aqueous alkaline environments
Source: Amino Acids. 2022 Dec 2;55(1):61–74. doi: 10.1007/s00726-022-03208-7 (PMC9877059; doi:10.1007/s00726-022-03208-7)
Supplement: Supplementary file 1 — Supplementary file1 (DOCX 2486 KB) [file 726_2022_3208_MOESM1_ESM.docx]

**Amino Acids**

**Electronic Supplementary Materials**

**Protein cysteine S-glycosylation: oxidative hydrolysis of protein S-glycosidic bonds in aqueous alkaline environments**

Alicja K. Buchowiecka

[alicja.buchowiecka@p.lodz.pl](mailto:alicja.buchowiecka@p.lodz.pl)

Institute of Molecular and Industrial Biotechnology

Lodz University of Technology

2/22 Stefanowskiego Street

50-537 Lodz, Poland

**Abstract**

Some glycoproteins contain carbohydrates S-linked to cysteine (Cys) residues. However, relatively few S-glycosylated proteins have been detected, due to the lack of an effective research methodology. This work outlines a general concept for the detection of S-glycosylation sites in proteins. The approach was verified by exploratory experiments on a model mixture of β-S-glucosylated polypeptides obtained by the chemical transformation of lysozyme P00698. The model underwent two processes: (1) oxidative hydrolysis of S-glycosidic bonds under alkaline conditions to expose the thiol group of Cys residues; (2) thiol S-alkylation leading to thiol S-adduct formation at the former S-glycosylation sites. Oxidative hydrolysis was conducted in aqueous urea, dimethyl sulfoxide, or trifluoroethanol, with silver nitrate as the reaction promoter, in the presence of triethylamine and/or pyridine. The concurrent formation of stable protein silver thiolates, gluconic acid, and silver nanoclusters was observed. The essential de-metalation of protein silver thiolates using dithiothreitol preceded the S-labeling of Cys residues with 4-vinyl pyridine or a fluorescent reagent. The S-labeled model was sequenced by tandem mass spectrometry to obtain data on the modifications and their distribution over the protein chains. This enabled the efficiency of both S-glycosidic bonds hydrolysis and S-glycosylation site labelling to be evaluated. Suggestions are also given for testing this novel strategy on real proteomic samples.

Keywords: proteomics, post-translational modifications, glycoproteins, S-linked glycosylation

**Table of Contents**

**I. Supplementary Figures and Supporting Comments**

**Fig. S1** β-Elimination reaction on Model D analyzed by thin layer chromatography. UV image of the TLC results. 4

**Fig. S2.** The deconvoluted ESI-MS spectrum of Model D. 5

**Fig. S3** The description of ESI-MS spectrum of Model D 6

**Fig. S4.** The assessment of the average S-glucosylation degree of Model D. Analysis of the T2, T3, and T4 groups of signals on the ESI-MS spectrum. 7

**Fig. S5.** The Byonic map visualizing the set of 320 unique peptides identified from Model D. 8

**Fig. S6.** UV/VIS absorption spectrum of the reaction mixture after oxidative hydrolysis of Model D by Procedure 1 (80% aq. DMSO/ NEt3; pH 9.1; 50°C; 120 min.) 9

**Fig. S7.** Oxidative hydrolysis of Model D by Procedure 4 (50 % aq. TFE/ NEt3 and Py; pH 8.6; 40°C; 150 min) monitored via UV/Vis absorption spectra. Processes of protein silver thiolates -C[S-Ag] and silver nanocluster-containing proteins -C[S-Ag@Ag] formation. 10

**Fig. S8**. Tryptic peptides originating from Model D. The distribution of S-Qat, Dha, S-Glc modifications over eight cysteine positions in polypeptides of Model D – based on CID/HCD data 11

**Fig. S9.** **PROCEDURE 1 -** fluorescent labeling of Model D. The distribituion of chemical modifi-cations over eight cysteine positions in the unique tryptic peptides identified by CID MS/MS. 12

**Fig. S10.** **PROCEDURE 1 –** re-bloking of cysteine thiols by APTA alkylation. The distribution of chemical modifications over eight cysteine positions in the unique tryptic peptides identified by CID MS/MS 13

**Fig. S11.** **PROCEDURE 1 –** re-bloking of cysteine thiols by APTA alkylation. The distribution of chemical modifications over eight cysteine positions in the unique tryptic peptides identified by HCD MS/MS 14

**Fig. S12.** **PROCEDURE 2 -** S-Glc sites labeling by pyridylethyl substituent (PE). The distribituion of chemical modifications over eight cysteine positions in the unique tryptic peptides identified by CID MS/MS. 15

**Fig. S13.** **PROCEDURE 2 -** S-Glc sites labeling by pyridylethyl substituent (PE). The distribution of chemical modifications over eight cysteine positions in the unique tryptic peptides identified by HCD MS/MS 16

**Fig. S14.** **PROCEDURE 3 -** S-Glc sites labeling by pyridylethyl substituent (PE). The distribution of chemical modifications over eight cysteine positions in the unique tryptic peptides identified by CiD MS/MS 17

**Fig. S15**. **PROCEDURE 3 -** S-Glc sites labeling by pyridylethyl substituent (PE). The distribution of chemical modifications over eight cysteine positions in the unique tryptic peptides identified by HCD MS/MS 18

**Fig. S16**. **PROCEDURE 4 -** S-Glc sites labeling by pyridylethyl substituent (PE). The distribution of chemical modifications over eight cysteine positions in the unique tryptic peptides identified by CID/HCD MS/MS 19

**Fig.S17.** CID spectrum of the S-glucosylated peptide **Y.SLGNWVC[+162]AAK.**F 20

**Fig.S18**. HCD spectrum of the S-glucosylated peptide **Y.SLGNWVC[+162]AAK**. 21

**Fig.S19.** CID spectrum of the S-glucosylated peptide **R.C[+162]KGTDVQAWIR**  22

**Fig.S20.** HCD spectrum of the S-glucosylated peptide **R.C[+162]KGTDVQAWIR.G** 23

**Table S1**. The statistical data of peptide mapping from Procedures 1- 4 versus data for Model D. 24

**Table S2**. Putative manifestations of reactive oxygen species activity in the tested Procedures 1-4 versus Model D. 24

**II. Collections of peptides identified by CID/HCD MS/MS sequencing _xlsx files**

**ESM 1_**Model D (5 sheets)

**ESM 2_**Procedure 1_BM-tagging (3 sheets)

**ESM 3_**Procedure 1.1_APTA re-blocking (6 sheets)

**ESM 4_**Procedure 2_PE-tagging (6 sheets)

**ESM 5_**Procedure 3_PE-tagging (6 sheets)

**ESM 6_**Procedure 4_PE-tagging (3 sheets)


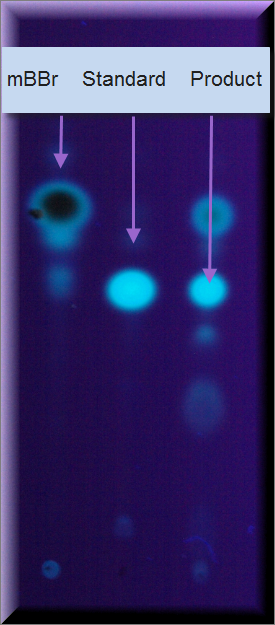


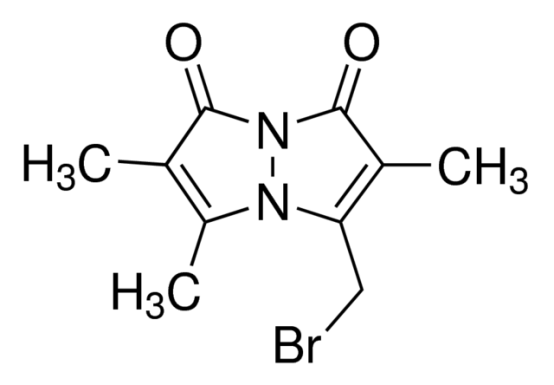


The structure of mBBr (monobromobimane),

the fluorescent thiol specific probe

**Fig. S1.** β-Elimination reaction on Model D analyzed by thin layer chromatography. UV image of the TLC results.

Reference samples: mBBr

Standard: BM-labeled 1-β-D-thioglucose

Analyte: Products of β-elimination reaction labeled with mBBr


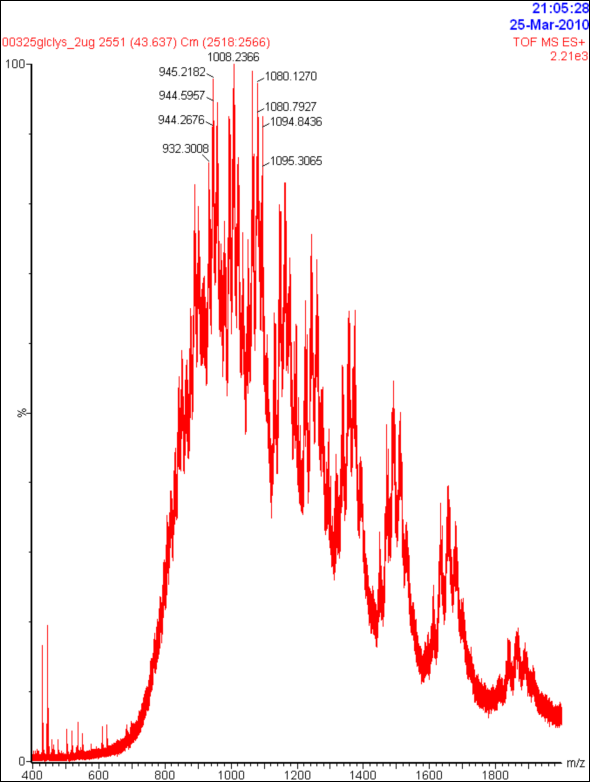

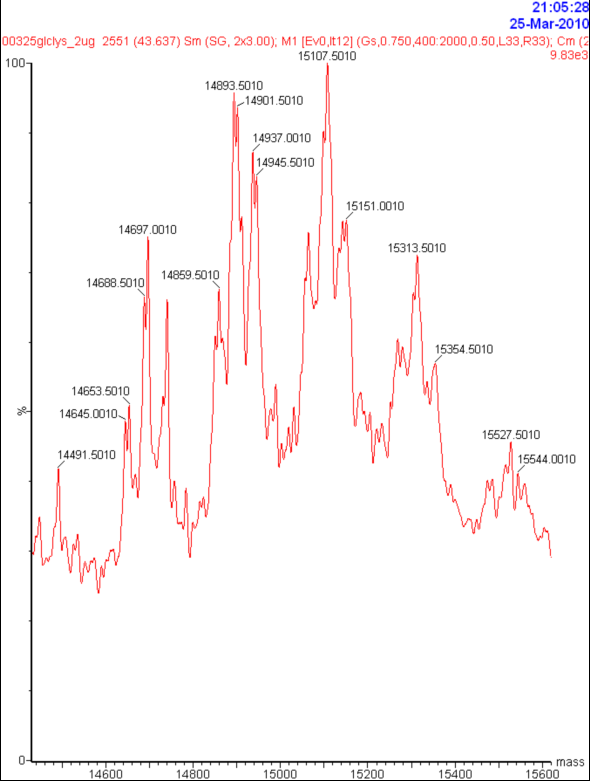


Deconvolution

„Triplets T” separated by *ca* 200 Da

**Fig. S2.** The deconvoluted ESI-MS spectrum of Model D.


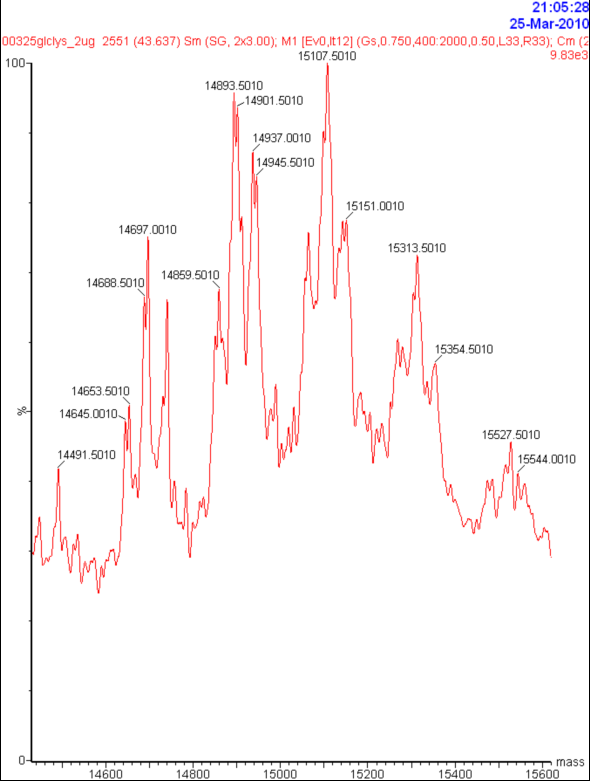


**Fig. S4.** The assessment of the average S-glucosylation degree of Model D.

**T2**

**T3**

**T4**

**T5**

**T6**

**T7**

|  | **T2** | **T3** | **T4** | **T5** | **T6** | **T7** |
| --- | --- | --- | --- | --- | --- | --- |
| Q+G (x +z) | 2 | 3 | 4 | 5 | 6 | 7 |
| Q (x) | 2 | 2 or 3 | 2 to 4 | 2 to 5 | 2 to 6 | 2 to 7 |
| G (z) | 0 | 0, 1 | 0, 1, 2 | 0, 1, 2, 3 | 0, 1, 2, 3, 4 | 0, 1, 2, 3, 4, 5 |
| Carbamy-lation | 0, 1, 2 | 0, 1, 2 | 0, 1, 2 | 0, 1, 2 | 0, 1, 2 | 0, 1, 2 |

**Fig. S4.** The description of the ESI-MS spectrum of Model D.

The S-glycosylated lysozyme is described by the general formula:

L-C[S-Qat]_x_-[Dha]_y_-C[S-Glc]_z_-[Side+]-COOH

This model forms a complex mixture of protein variants obtained as a result of adding 1-β-D-thio-glucose to Dha residues in the precursor assigned as:

L-C[S-Qat] **_2 ± 1_**-[Dha] **_6 ± 1_**-[Side+]-COOH

The molecule bearing no side modifications and described by the general formula L-C[S-Qat]_x_-[Dha]_y_-[COOH] served as a starting point for interpreting the deconvo-luted ESI-MS spectrum of Model D. The theoretical molecular masses for structural variants of L-C[S-Qat]x-[Dha]y-[COOH] differing by x and y values from the allowed range from 0 to 8 were calculated. The relevant data and results present the following tables.

| **Symbol** | **[Da]** |
| --- | --- |
| Qat : **Q** | 170.1419 |
| Glc : **G** | 162.0528 |
| Qat-SH | 203.1218 |
| Glc_SH | 195.0327 |
| **Q - G** | 8.08908  **8.1** |

| L-C[S-Qat]_x_-[Dha]_y_-[COOH] | | |
| --- | --- | --- |
| x | y | m/z calc. [Da] |
| 8 | 0 | 15673.08 |
| 7 | 1 | 15467.94 |
| 6 | 2 | 15262.80 |
| 5 | 3 | 15057.67 |
| 4 | 4 | 14852.53 |
| 3 | 5 | 14647.39 |
| 2 | 6 | 14442.25 |
| 1 | 7 | 14237.12 |
| 0 | 8 | 14031.98 |

The central signal of T2 “triplet” observed at 14491.5 m/z can be assigned to the proteins of isobaric compositions:

L-C[S-Qat]_2_-[Dha]_6_-[Side+]-[COOH].

The calculated difference: 14491.5 (obs.) - 14442.3 (calc.) = 49.3 Da corresponds to [Side+] modifications of proteins carrying the single carbamylation (43 Da) and six deamidations on Asn/Gln sites (6 Da). The lower intensity signals, shifted by 43 Da to the left or right side from the 14491.5 m/z, can be assigned to structures, respectively, non-carbamylated and doubly carbamylated.

**Fig. S3.** The description of the ESI-MS spectrum of Model D.

**Calculations for T2**

14491.5 (*obs.*) - 14442.3 (*calc.*) = 49.3 Da – 43 Da (c*arbamylation*) = 6.3 Da (*[Side+]*)

**Calculations for T3**

14491.5 Da (*obs.*) + 203.1 Da (*Qat-SH*) = 14694.6 Da (*calc.*) 14697.0 Da (*obs.*)

14491.5 Da (*obs.*) + 195.0 Da (*Glct-SH*) = 14686.5 Da (*calc.*) 14688.5 Da (*obs.*)

The group of T3 signals looks like two overlying triplets shifted by around 8 Da. Interpreting that feature requires the theoretical addition of 0.5 equivalent of Qat-SH and 0.5 equivalent of Glc-SH to a single Dha residue existing in proteins hidden under T2 triplet.

**Calculations for T4**

14694.6 Da (*obs.*) + 203.1 Da (*Qat-SH*) = 14897.7 Da (*calc.*) 14901.5 Da (*obs.*)

14694.6 Da (*obs.*) + 195.0 Da (*Glct-SH*) = 14889.6 Da (*calc.*) 14893.5 Da (*obs.*)

14686.5 Da (*obs.*) + 203.1 Da (*Qat-SH*) = 14889.6 Da (*calc.*) 14893.5 Da (*obs.*)

14686.5 Da (*obs.*) + 195.0 Da (*Glct-SH*) = 14881.5 Da (*calc.*) (*hidden signal*)

The same reasoning applied to the T4 group of signals, which exhibit the double 8 Da splittings, indicates isobaric proteins of the S-glucosylation degree: 0, 1, 2.

The same type of calculations were done for the T5, T6, and T7 groups of signals. The character-ristics of their profiles do not permit the detailed

Similar calculations applied to the T5, T6, and T7 groups of signals did not permit detailed interpretation of this section of ESI-MS spectrum. This part of the spectrum profile is less resolved. It represents the isobaric groups of polypeptides that differ by side modifications, such as deamidations and others.

In conclusion, the T3, T4, T5 groups of signals as most intense served for the assess-ment of the average S-glucosylation degree of Model D. T3, T4 and T5 signals represents proteins carrying respectively up to 1, 2, and 3 D-Glc residues per molecule. Therefore, the average S-glucosylation level was accepted as 2 ± 1.

**Fig. S4.** The assessment of the average S-glucosylation degree of Model D. Analysis of the T2, T3, and T4 groups of signals on the ESI-MS spectrum.


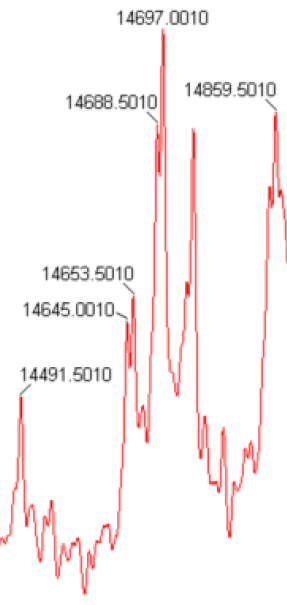


**QQG**

**QQQ**

Q - G = 8 Da

Carbamylation (+43 Da)

0 1 2 3

43 Da

**T3**


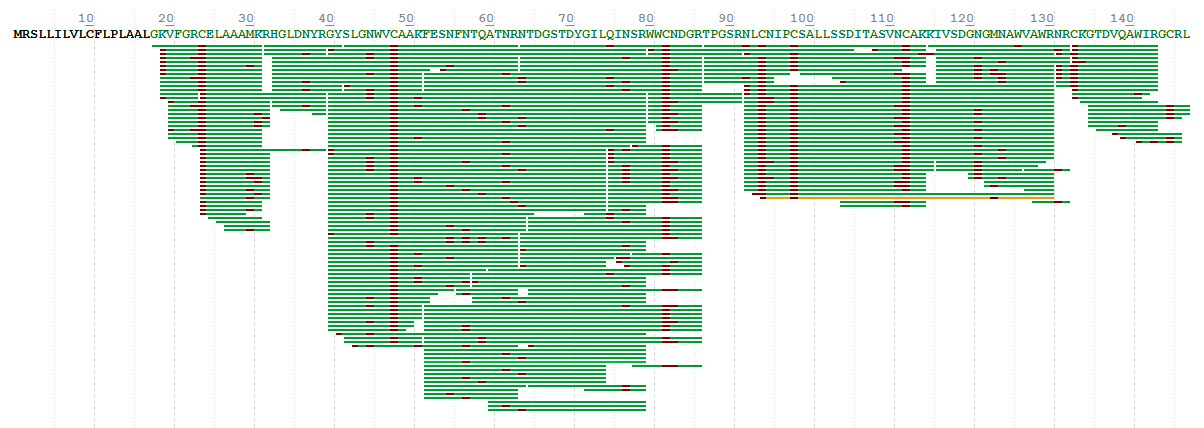


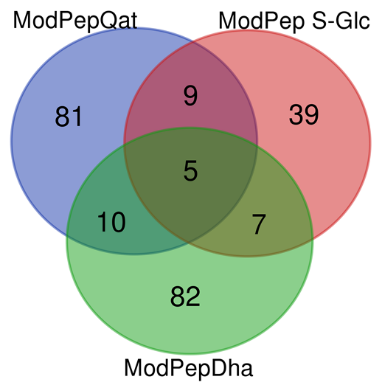
**Fig. S5.** The Byonic map visualizing the set of 320 unique peptides identified from Model D.

The Byonic map starts with the sequence of Lysozyme P00698 preprotein containing 147 amino acid residues ( 18 aa residues of the signal peptide are marked in black). 320 unique peptides are depicted as green lines with red marks indicating sites of divers modifications. The Venn diagram represents correlations between the subsets of model peptides carrying three types of modifications chemically introduced into cysteine positions: Qat (quaternary amine), S-Glc (S-glucosylation), Dha (dehydroalanine). Five large peptides are bearing each of these modifications within a single sequence. (<http://bioinformatics.psb.ugent.be/webtools/Venn/>)

**Fig. S6.** UV/VIS absorption spectrum of the reaction mixture after oxidative hydrolysis of Model D by Procedure 1 (80% aq. DMSO/ NEt3; pH 9.1; 50°C; 120 min.)

**Fig. S7.** Oxidative hydrolysis of Model D by Procedure 4 (50 % aq. TFE/ NEt3 and Py; pH 8.6; 40°C; 150 min) monitored via UV/Vis absorption spectra. Processes of protein silver thiolates -C[S-Ag] and silver nanocluster-containing proteins -C[S-Ag@Ag] formation.

| C pos. in | No & type of modifications_ Model D | | | | | CID/HCD |
| --- | --- | --- | --- | --- | --- | --- |
| P00698 | C[S-Qat] | Dha | C[S-Glc] | C[SO_3_H] | C[S-H] | total. |
| 6 | 16 | 15 | 11 | 0 | 1 | 43 |
| 30 | 20 | 50 | 2 | 0 | 4 | 76 |
| 64 | 29 | 15 | 14 | 0 | 2 | 60 |
| 76 | 15 | 6 | 8 | 1 | 1 | 31 |
| 80 | 12 | 7 | 10 | 0 | 0 | 29 |
| 94 | 14 | 7 | 9 | 0 | 3 | 33 |
| 115 | 8 | 0 | 7 | 0 | 1 | 16 |
| 127 | 0 | 1 | 1 | 2 | 1 | 5 |
| total | 114 | 101 | 62 | 3 | 13 | 293 |

**Fig.S8**. The distribution of S-Qat, Dha, S-Glc modifications over eight cysteine positions in polypeptides of Model D – based on CID/HCD data.

**Table S8**. Numeric data for the diagram in Fig.S8

**Fig. S9**. The distribution of chemical modifications over eight cysteine positions in the unique tryptic peptides identified by CID MS/MS.

**Table S9**. Numeric data for the diagram in Fig.S9

| C pos. in | No and type of modifications at a particular cysteine position | | | | | | CID |
| --- | --- | --- | --- | --- | --- | --- | --- |
| P00698 | C[S-Qat] | Dha | C[S-Glc] | C[SO_3_H] | C[S-H] | C[S-BM] | total |
| 6 | 26 | 25 | 10 | 2 | 1 | 3 | 67 |
| 30 | 40 | 37 | 4 | 1 | 5 | 5 | 92 |
| 64 | 45 | 12 | 6 | 1 |  | 4 | 68 |
| 76 | 67 | 35 | 32 | 4 | 11 | 6 | 155 |
| 80 | 57 | 72 | 29 | 3 | 17 | 6 | 184 |
| 94 | 68 | 49 | 19 | 1 | 20 | 22 | 179 |
| 115 | 14 | 4 | 7 | 1 | 1 | 1 | 28 |
| 127 |  | 3 | 2 |  | 1 |  | 6 |
| total | 317 | 237 | 109 | 13 | 56 | 47 | 779 |

**Fig. S10.** The distribution of chemical modifications over eight cysteine positions in the unique tryptic peptides identified by CID MS/MS.

**Table S10**. Numeric data for the diagram in Fig. S10

| C pos. in | No and type of modifications at a particular cysteine position | | | | | CID |
| --- | --- | --- | --- | --- | --- | --- |
| P00698 | C[S-Qat] | Dha | C[S-Glc] | C[SO_3_H] | C[S-H] | total |
| 6 | 39 | 26 |  |  |  | 65 |
| 30 | 38 | 50 |  |  |  | 88 |
| 64 | 38 | 8 |  |  |  | 46 |
| 76 | 68 | 38 | 28 | 6 | 8 | 148 |
| 80 | 63 | 49 | 32 | 6 | 2 | 152 |
| 94 | 65 | 87 | 13 |  | 5 | 170 |
| 115 | 12 | 2 |  |  |  | 14 |
| 127 | 4 | 2 |  |  |  | 6 |
| total | 327 | 262 | 73 | 12 | 15 | 689 |

**Fig. S11**. The distribution of chemical modifications over eight cysteine positions in the unique tryptic peptides identified by HCD MS/MS.

**Table S11**. Numeric data for the diagram in Fig.S11

| C pos. in | No and type of modifications at a particular cysteine position | | | | | **HCD** |
| --- | --- | --- | --- | --- | --- | --- |
| P00698 | C[S-Qat] | Dha | C[S-Glc] | C[SO_3_H] | C[SH] | total |
| 6 | 37 | 23 | 8 | 2 |  | 70 |
| 30 | 44 | 36 | 4 |  |  | 84 |
| 64 | 38 | 11 | 6 |  |  | 55 |
| 76 | 52 | 22 | 19 |  | 3 | 96 |
| 80 | 39 | 28 | 23 | 2 | 6 | 98 |
| 94 | 42 | 46 | 9 |  | 10 | 107 |
| 115 | 6 | 3 | 5 |  | 1 | 15 |
| 127 | 17 | 3 | 2 |  |  | 22 |
| total | 275 | 172 | 76 | 4 | 20 | 547 |

**Fig. S12.** The distribution of chemical modifications over eight cysteine positions in the unique tryptic peptides identified by CID MS/MS.

**Table S12**. Numeric data for the diagram in Fig.S12

| C pos. in | No and type of modifications at a particular cysteine position | | | | | | CID |
| --- | --- | --- | --- | --- | --- | --- | --- |
| P00698 | C[S-Qat] | Dha | C[S-Glc] | C[SO_3_H] | C[S-H] | C[S-PE] | total |
| 6 | 9 | 17 | 5 |  |  | 2 | 33 |
| 30 | 14 | 44 | 2 |  |  | 2 | 62 |
| 64 | 18 | 15 | 5 | 1 | 1 | 5 | 45 |
| 76 | 20 | 9 | 3 | 5 | 19 | 10 | 66 |
| 80 | 10 | 15 | 12 | 2 | 18 | 10 | 67 |
| 94 | 12 | 19 | 2 | 3 | 19 | 13 | 68 |
| 115 | 4 | 4 | 7 | 1 |  | 3 | 19 |
| 127 | 3 | 1 | 1 |  |  |  | 5 |
| total | 90 | 124 | 37 | 12 | 57 | 45 | 365 |

**Fig. S13.** The distribution of chemical modifications over eight cysteine positions in the unique tryptic peptides identified by HCD MS/MS

**Table S13**. Numeric data for the diagram in Fig. S13

| C pos. in | No and type of modifications at a particular cysteine position | | | | | | HCD |
| --- | --- | --- | --- | --- | --- | --- | --- |
| P00698 | C[S-Qat] | Dha | C[S-Glc] | C[SO_3_H] | C[S-H] | C[S-PE] | total |
| 6 | 12 | 20 | 3 | 3 |  | 3 | 41 |
| 30 | 21 | 24 | 2 |  |  | 2 | 49 |
| 64 | 18 | 20 | 4 | 1 | 2 | 6 | 51 |
| 76 | 10 | 11 | 5 | 1 | 4 | 5 | 36 |
| 80 | 7 | 6 | 3 |  | 6 | 6 | 28 |
| 94 | 9 | 14 | 3 | 1 | 9 | 3 | 39 |
| 115 | 3 | 2 | 5 | 2 |  | 3 | 15 |
| 127 | 3 | 2 | 1 |  |  |  | 6 |
| total | 83 | 99 | 26 | 8 | 21 | 28 | 265 |

**Fig. S14.** The distribution of chemical modifications over eight cysteine positions in the unique tryptic peptides identified by CID MS/MS

**Table S14.** Numeric data for the diagram in Fig. S14

| C pos. in | No and type of modifications at a particular cysteine position | | | | | | CID |
| --- | --- | --- | --- | --- | --- | --- | --- |
| P00698 | C[S-Qat] | Dha | C[S-Glc] | C[SO_3_H] | C[S-H] | C[S-PE] | total |
| 6 | 4 | 12 | 4 | 5 |  |  | 31 |
| 30 | 7 | 25 | 20 | 1 | 2 | 4 | 89 |
| 64 | 19 | 31 | 5 | 25 | 13 | 3 | 160 |
| 76 | 24 | 18 | 19 | 26 | 28 | 22 | 213 |
| 80 | 16 | 20 | 21 | 27 | 27 | 26 | 217 |
| 94 | 10 | 18 | 23 | 32 | 43 | 17 | 237 |
| 115 | 3 | 1 | 7 | 7 | 1 | 1 | 135 |
| 127 |  |  | 1 | 1 | 1 |  | 130 |
| total | 83 | 125 | 100 | 124 | 115 | 73 | 1212 |

**Fig. S15.** The distribution of chemical modifications over eight cysteine positions in the unique tryptic peptides identified by HCD MS/MS

**Table S15.** Numeric data for the diagram in Fig. S15

| C pos. in | No and type of modifications at a particular cysteine position | | | | | | HCD |
| --- | --- | --- | --- | --- | --- | --- | --- |
| P00698 | C[S-Qat] | Dha | C[S-Glc] | C[SO_3_H] | C[S-H] | C[S-PE] | total |
| 6 | 5 | 8 | 2 |  |  |  | 15 |
| 30 | 8 | 14 |  |  |  |  | 22 |
| 64 | 17 | 22 | 13 | 1 | 6 | 3 | 62 |
| 76 | 1 | 2 | 1 |  |  | 1 | 5 |
| 80 | 8 |  | 2 |  |  | 1 | 11 |
| 94 | 1 | 1 | 1 |  | 1 | 4 | 8 |
| 115 | 2 | 1 | 6 | 1 |  | 2 | 12 |
| 127 |  | 1 | 1 |  |  | 2 | 4 |
| total | 42 | 49 | 26 | 2 | 7 | 13 | 133 |

**Fig. S16.** The distribution of chemical modifications over eight cysteine positions in the unique tryptic peptides identified by CID/HCD MS/MS

**Table S16.** Numeric data for the diagram in Fig. S16

| C pos. in | No and type of modifications at a particular cysteine position | | | | | | CID/HCD |
| --- | --- | --- | --- | --- | --- | --- | --- |
| P00698 | C[S-Qat] | Dha | C[S-Glc] | C[SO_3_H] | C[S-H] | C[S-PE] | total |
| 6 |  |  | 10 |  | 2 | 1 | 13 |
| 30 |  |  | 6 |  |  | 4 | 10 |
| 64 |  | 2 | 18 |  | 3 | 1 | 24 |
| 76 | 16 | 9 | 17 | 5 | 18 | 14 | 79 |
| 80 | 18 | 9 | 15 | 1 | 17 | 17 | 77 |
| 94 | 18 | 13 | 11 | 2 | 22 | 11 | 77 |
| 115 |  |  | 5 |  |  | 1 | 6 |
| 127 |  |  | 1 |  |  | 1 | 2 |
| total | 52 | 33 | 83 | 8 | 62 | 50 | 288 |


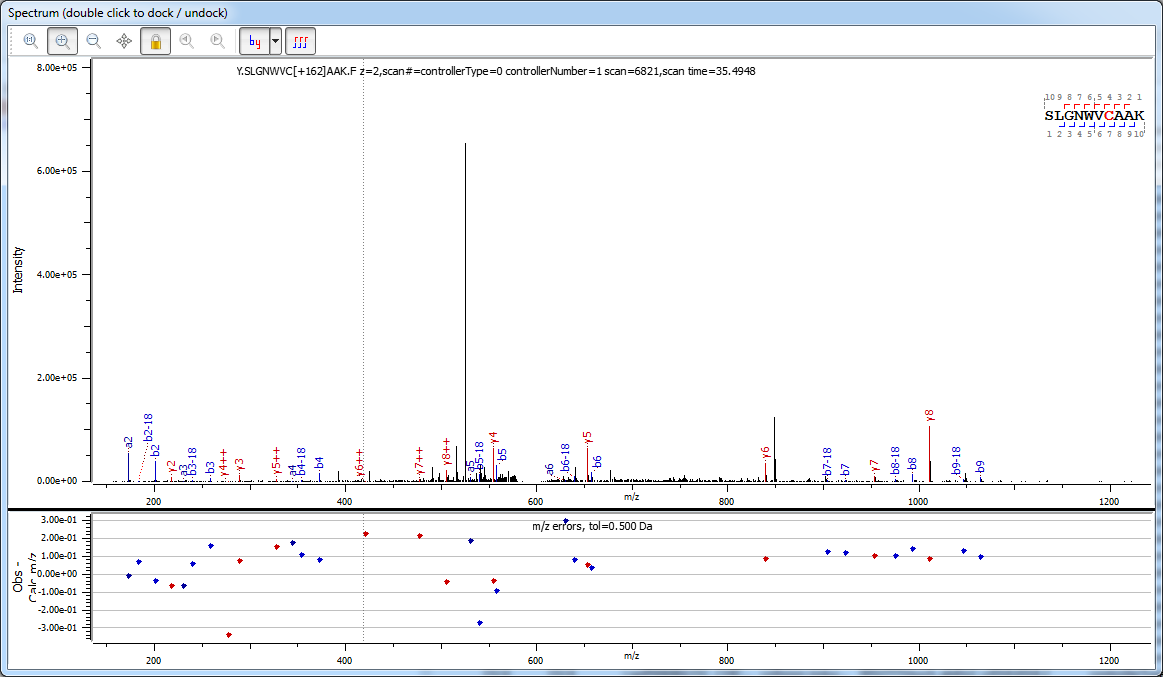


**1048.6554**

**524.8865**

**Figure. S17.** CID spectrum of the peptide **Y.SLGNWVC[+162]AAK.F** (scan time 35.4948)

| **Peptide number** | **Calc. MW** | **Score** | **z** | **Calc. NL from parent ion**  **[MHz-162]/z** | **Obs. NL from parent ion**  **[MHz-162]/z** | **Intensity** | **Calc. NL from molecular ion**  **[MH-162]+** | **Obs. NL from molecular ion**  **[MH-162]+** | **Intensity** |
| --- | --- | --- | --- | --- | --- | --- | --- | --- | --- |
| 10 | 1209.5699 | 377.6 | +2 | 524.7923 | 524.8865 | 6.53E+05 | 1048.57718 | 1048.6554 | 1.47E+04 |


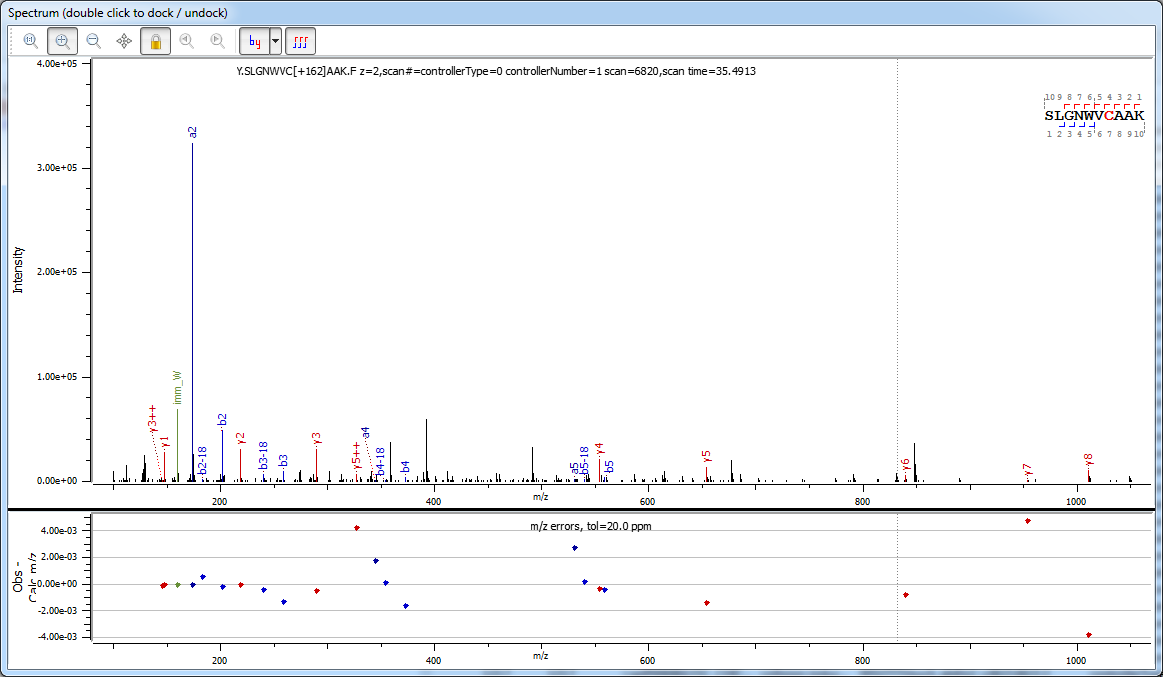


**1048.5214**

**Figure. S18.** HCD spectrum of the peptide **Y.SLGNWVC[+162]AAK.F** (scan time 35.4913)

| **Peptide number** | **Calc. MW** | **Score** | **z** | **Calc. NL from parent ion**  **[MHz-162]/z** | **Obs. NL from parent ion**  **[MHz-162]/z** | **Intensity** | **Calc. NL from molecular ion**  **[MH-162]+** | **Obs. NL from molecular ion**  **[MH-162]+** | **Intensity** |
| --- | --- | --- | --- | --- | --- | --- | --- | --- | --- |
| 10 |  | 407.9 | +2 | 524.7659 | Not observed | NA | 1048.5244 | 1048.5214 | 4.47E+03 |


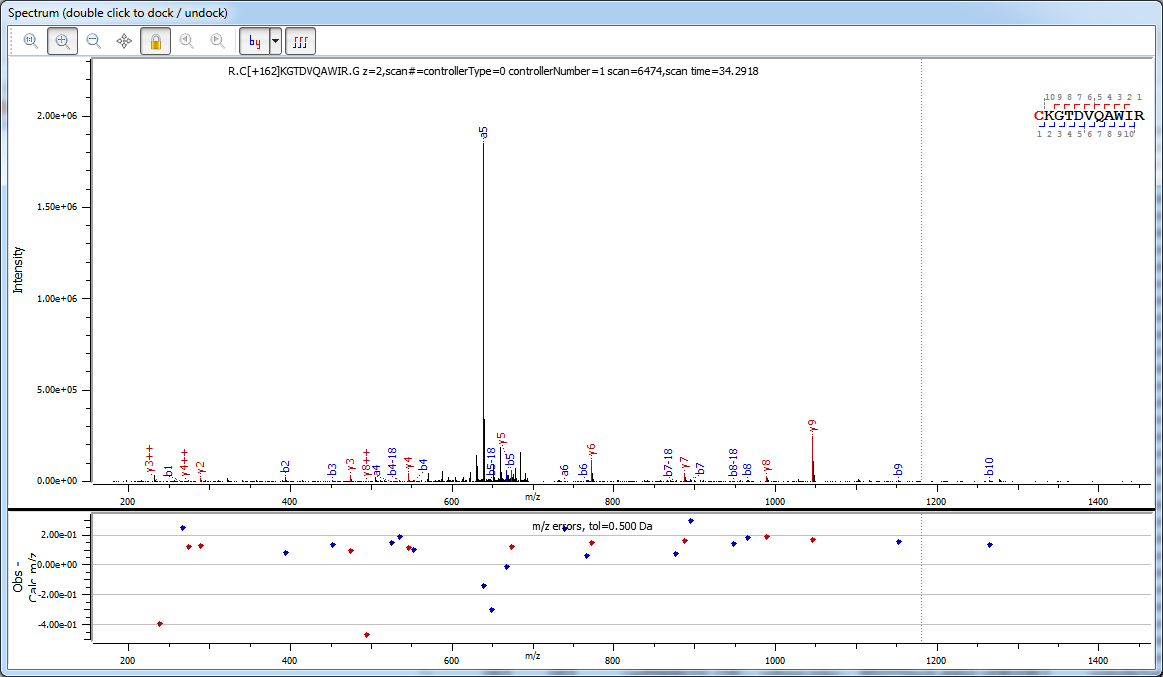


**639.1299**

**1276.7574**

**Figure. S19.** CID spectrum of the peptide **R.C[+162]KGTDVQAWIR.G** (scan time 34.2918)

| **Peptide number** | **Calc. MW** | **Score** | **z** | **Calc. NL from parent ion**  **[MHz-162]/z** | **Obs. NL from parent ion**  **[MHz-162]/z** | **Intensity** | **Calc. NL from molecular ion**  **[MH-162]+** | **Obs. NL from molecular ion**  **[MH-162]+** | **Intensity** |
| --- | --- | --- | --- | --- | --- | --- | --- | --- | --- |
| 36 | 1437.6922 | 354.2 | +2 | 638.8534 | 639.1299 | 1.85E+06 | 1276.69948 | 1276.7574 | 1276.7574 |


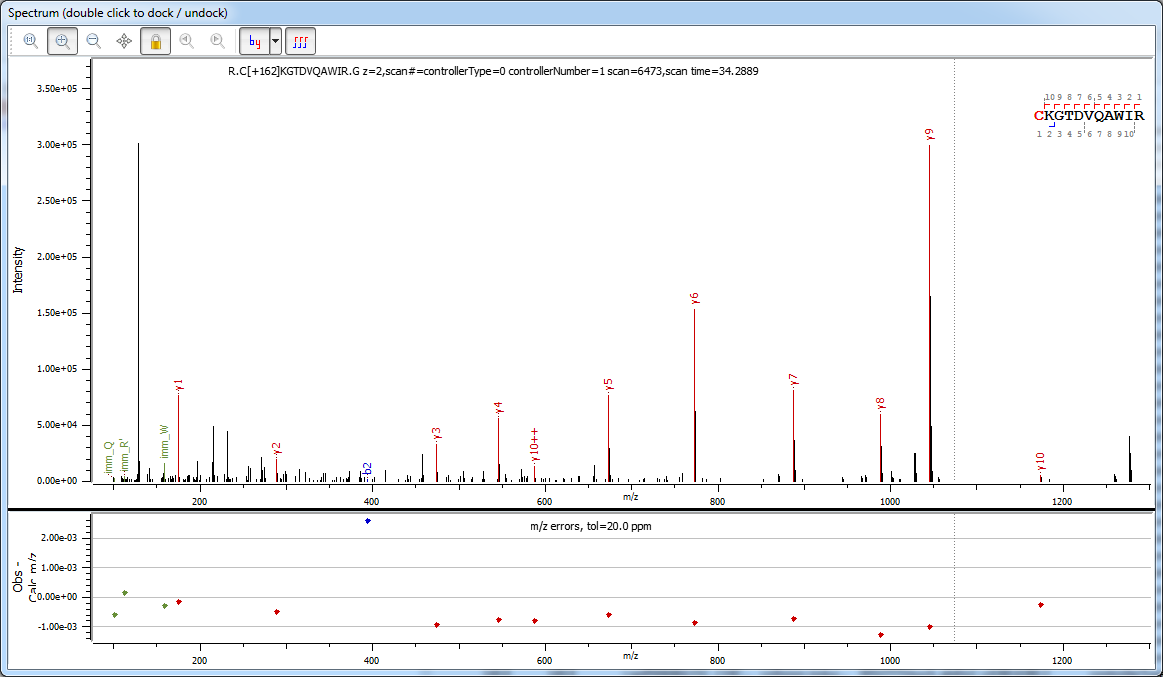


**683.8270**

**1276.6472**

**Figure. S20.** HCD spectrum of the peptide **R.C[+162]KGTDVQAWIR.G** (scan time 34.2889)

| **Peptide number** | **Calc. MW** | **Score** | **z** | **Calc. NL from parent ion**  **[MHz-162]/z** | **Obs. NL from parent ion**  **[MHz-162]/z** | **Intensity** | **Calc. NL from molecular ion**  **[MH-162]+** | **Obs. NL from molecular ion**  **[MH-162]+** | **Intensity** |
| --- | --- | --- | --- | --- | --- | --- | --- | --- | --- |
| 36 | 1437.6922 | 388.6 | +3 | 638.8272 | 638.8270 | 4.87E+03 | 1276.6467 | 1276.6472 | 3.99E+04 |

**Table S1**. The statistical data of peptide mapping from Procedures 1- 4 versus data for Model D.

| peptides | Model D | S-Glc bond hydrolysis in the model D and the subsequent S-tagging | | | | | | | |
| --- | --- | --- | --- | --- | --- | --- | --- | --- | --- |
| *(* peps.) | 8 M urea | **Procedure 1**_DMSO (aq) | | | **Procedure 2**_DMSO(aq)/urea | | **Procedure 3**_Urea (aq) | | **Proc.4**_TFE (aq) |
| **Number of** | CID-HCD | APTA/CID | APTA/HCD | mBBr/CID | 4-VP/CID | 4-VP/HCD | 4-VP/CID | 4-VP/HCD | 4-VP/CID-HCD |
| spectra | 3319 | 6323 | 7256 | 6272 | 3337 | 2298 | 1728 | 1098 | 4359 |
| unique peps | 313 | 526 | 475 | 591 | 335 | 295 | 449 | 221 | 490 |
| modified peps. | 280 | 477 | 420 | 544 | 304 | 262 | 410 | 188 | 459 |
| Cys-cont. peps. | 244 | 411 | 369 | 185 | 193 | 162 | 259 | 117 | 389 |
| S-tagged peps. | 105 | 213 | 209 | 47 | 39 | 25 | 54 | 6 | 41 |
| C[SH] + C[SO3H] | 14 | 21 | 20 | 50 | 47 | 20 | 115 | 9 | 53 |
| C[S-Glc] | 60 | 64 | 70 | 100 | 37 | 29 | 89 | 25 | 74 |

**Table S2.** Putative manifestations of reactive oxygen species activity in the tested Procedures 1-4 versus Model D.

| peptides | Model D | S-Glc bond hydrolysis in the model D - oxidation phenomenon via ROS | | | | | | | |
| --- | --- | --- | --- | --- | --- | --- | --- | --- | --- |
| (peps.) | 8 M urea | **Procedure 1**_DMSO (aq) | | | **Procedure 2**_DMSO(aq)/urea | | **Procedure 3**_Urea (aq) | | **Proc.4**_TFE (aq) |
| **Number of** | CID-HCD | APTA/CID | APTA/HCD | mBBr/CID | 4-VP/CID | 4-VP/HCD | 4-VP/CID | 4-VP/HCD | 4-VP/CID-HCD |
| spectra | 3319 | 6323 | 7256 | 6272 | 3337 | 2298 | 1728 | 1098 | 4359 |
| unique peps | 313 | 526 | 475 | 591 | 335 | 295 | 449 | 221 | 490 |
| modified peps. | 280 | 477 | 420 | 544 | 304 | 262 | 410 | 188 | 459 |
| **ROS oxidation** | Number of identified Cysteine-, Methionine- and Tryptophane-containing peptides oxidized by ROS | | | | | | | | |
| M[+16] peps. | 9 | 24 | 22 | 5 | 15 | 14 | 0 | 7 | 23 |
| M[+32] peps. | 2 | 1 | 2 | 5 | 0 | 2 | 10 | 1 | 2 |
| W[+32] peps. | 2 | 0 | 5 | 5 | 1 | 1 | 16 | 4 | 13 |
| C[+48] peps | 3 | 13 | 4 | 13 | 12 | 6 | 113 | 2 | 8 |
